# Mediative role of body mass index in cardiorespiratory fitness-associated vascular remodeling in youth

**DOI:** 10.1038/s41390-024-03589-3

**Published:** 2024-09-18

**Authors:** Luisa Semmler, Lisa Baumgartner, Heidi Weberruß, Raphael Pirzer, Renate Oberhoffer-Fritz

**Affiliations:** 1 Department of Neurology, Klinikum rechts der Isar, Technical University Munich, Munich, Germany; 2 Institute of Preventive Pediatrics, Technical University of Munich, Munich, Germany; 3 Clinic for Pediatric and Adolescent Medicine, Klinikum Bayreuth, Bayreuth, Germany; 4 Department of Anaesthesiology and Operative Intensive Care, University Hospital Augsburg, Augsburg, Germany

## Abstract

**Background:**

Data on fitness-associated arterial remodeling in children is limited. We assessed the relation between cardiorespiratory fitness (CRF) and intima-media thickness (IMT), diameter, IMT:diameter-ratio (IDR), and tensile stress of the common carotid artery (CCA) in 697 healthy German schoolchildren. Further, we explored how body mass index (BMI) may influence these associations.

**Methods:**

We measured the vascular parameters with a high-resolution ultrasound device. We determined CRF using the FITNESSGRAM® PACER test and calculated each child’s allometrically scaled peak oxygen uptake capacity (VO_2_peak).

**Results:**

VO_2_peak, reflecting CRF, showed positive direct effects on IMT (girls: *p* < 0.001; boys: *p* = 0.02) and diameter in girls (*p* < 0.001). Considering BMI as a mediator, higher CRF was indirectly linked to decreases in IMT (girls: *p* = 0.04; boys: *p* = 0.02) and diameter (both *p* < 0.001), reflecting a competitive mediation. CRF indirectly mitigated the BMI-associated decrease in IDR (both *p* < 0.001) and increase in tensile stress (both *p* < 0.001) without affecting any of these parameters directly.

**Conclusion:**

CRF appears to be linked to uniform arterial remodeling with balanced hemodynamics and to further alleviate BMI-associated, potentially adverse vascular alterations, highlighting its significant role in cardiovascular health in youth.

**Impact:**

- Data on CRF-associated arterial remodeling in youth is limited.
- Higher VO_2_peak, reflecting higher CRF, was positively associated with IMT in girls and boys and diameter in girls. These direct effects were counteracted by the indirect BMI-mediated effect of CRF on IMT and diameter, reflecting a competitive mediation.
- A higher CRF indirectly mitigated the BMI-associated decrease in IDR and increase in tensile stress without directly affecting any of these parameters.
- Our findings indicate homogenous remodeling and balanced hemodynamics with increasing CRF—and opposite effects with increasing BMI.

## Introduction

Cardiovascular disease (CVD) accounts for approximately one-third of all deaths worldwide.^1^ Some known risk factors already occur in childhood, e.g., obesity,^2^ dyslipidemia,^3^ high blood pressure,^4^ endothelial dysfunction,^5^ and even atherosclerosis as a manifest CVD-associated disease.^6^ The ultrasound assessment of intima-media thickness (IMT) has been suggested as a non-invasive surrogate marker for subclinical atherosclerosis in children.^7^ In fact, increased IMT has been found in children with obesity,^8^ hypertension,^9^ familial hypercholesterolemia,^10^ type 1 diabetes,^11^ non-alcoholic fatty liver disease,^12^ and chronic kidney disease.^13^ In adults, a higher IMT is associated with an increased risk of myocardial infarction and stroke.^14,15^

On the other hand, a known protective factor against CVD is cardiorespiratory fitness (CRF).^16^ Higher CRF has been associated with reduced CVD-dependent morbidity and mortality.^17,18^ Theoretically, IMT would be expected to be lower in children with higher CRF. However, there is conflicting evidence with studies describing an increased IMT in healthy, fit children and adolescent athletes compared to controls.^19–23^ Weberruß et al. showed that despite an increased IMT, the carotid arteries of fit children had higher arterial compliance and lower stiffness, indicating improved arterial function.^24^ This raises the question of whether a higher IMT in healthy, fit children reflects physiological adaptation rather than subclinical atherosclerosis. Besides IMT, further parameters such as the arterial diameter and the IMT:diameter-ratio (IDR) determine a vessel’s geometry.^25^ These parameters change in specific ways depending on the underlying trigger of arterial remodeling – pathological or physiological. For example, atherosclerosis is classically described as an outward hypertrophic remodeling with a thickening of the wall accompanied by an increase in IDR.^25^ Aneurysm formation is characterized by outward hypotrophic remodeling, meaning an increase in vessel diameter, that goes along with a thinning of the vessel wall and results in a reduced IDR.^25^ Arterial structure changes through hypertension are caused by inflammation, apoptosis, and vascular fibrosis and are characterized mainly as hypertrophic remodeling: outer and lumen diameter are reduced, and media/lumen ratio is increased.^26^ In contrast, an athlete’s artery is deemed a vessel with decreased IMT and IDR but increased diameter.^27^ However, little is known about the detailed fitness-associated remodeling processes, especially in children.

CRF is in part genetically determined but is also subject to environmental influences, predominantly exercise: a planned, structured, repetitive bodily activity.^16^ Exercise seems to cause changes in the hemodynamic forces shear stress and tensile stress.^28^ Shear stress is the tangential force of the flowing blood on the endothelial surface, and tensile stress is the circumferential wall tension divided by wall thickness, which acts perpendicularly on the arterial wall.^29,30^ Changes in these hemodynamic forces may lead to the activation of the so-called mechanotransduction cascade. Thereby, shear and tensile stress alterations trigger specialized cells in the vascular wall, activating biological downstream signaling pathways that regulate gene expression and ultimately leading to vascular restructuring.^28^ In addition to the direct influence of CRF and exercise on the vasculature, indirect effects might occur through accompanying changes in body composition. For example, CRF is inversely related to body mass index (BMI)^31^, and BMI, then again, is thought to influence IMT and vascular diameter.^8,32^

Examining diameter and IDR in addition to IMT and considering indirect fitness-related effects on body composition could provide deeper insights into arterial remodeling processes in children and thus help differentiate between children with and without CVD risk. Therefore, the aim of our study was firstly to investigate the influence of CRF on IMT, diameter, IDR, and tensile stress of the common carotid artery (CCA) in 697 healthy German schoolchildren and secondly, to clarify the role of the indirect effect mediated by BMI.

## Methods

### Study design and population

This cross-sectional observational study enrolled 1017 healthy schoolchildren aged 7–18 years. Data were collected from October 2012 to July 2013 as part of the “Sternstunden der Gesundheit” project in the Berchtesgadener Land region of Germany.^23^

Written informed consent was obtained from all children aged ≥14 years and all participants’ parents. The study was approved by the local ethics committee (5490/12) of the Technical University of Munich and met the ethical guidelines of the Declaration of Helsinki (revised version 2013). Children were examined by trained staff.

### Measurements

#### Anthropometry

Body mass and height were measured to the nearest 0.1 kg and 0.1 cm (seca 799; Seca, Hamburg, Germany) without shoes. BMI was calculated as follows^33^:

$${{{\rm{BMI}}}}=\frac{{{{\rm{body\; mass}}}}}{{{{\rm{hei}}}}{{{{\rm{ght}}}}}^{2}}\left[\frac{{{{\rm{kg}}}}}{{{{{\rm{m}}}}}^{2}}\right]$$

We regarded children with a BMI ≥ 97th percentile as obese and those with a BMI ≥ 90th percentile as overweight.^34^

#### Blood pressure

Peripheral systolic and diastolic blood pressure (SBP, DBP) were measured on the left upper arm with an oscillometric device (Mobil-O-Graph; I.E.M., Stolberg, Germany) after the participant rested for 10 min. Mean arterial blood pressure (MAP) was calculated as follows^35^:

$${{{\rm{MAP}}}}=\frac{{{{\rm{SBP}}}}+2 * {{{\rm{DBP}}}}}{3}\left[{{{\rm{mmHg}}}}\right]$$

#### Vascular parameters

IMT and diameter of the CCA were assessed utilizing semi-automated B- and M-Mode ultrasound on the ProSound Alpha 6 system (Aloka/Hitachi Medical Systems GmbH, Wiesbaden, Germany). This approach combines automatic edge detection with manual correction, employing a high-frequency linear array probe (5–13 MHz). After 15 min of rest, children underwent the examination in the supine position, with the neck slightly extended and the head turned 45 degrees opposite the scanned site. IMT measurement in B-Mode adhered to the Mannheim Consensus,^36^ focusing on the CCA far wall, 1 cm proximal to the bulb at the end-diastolic moment (R-wave) when IMT is thickest. The cardiac cycle was recorded with a three-lead ECG. Four measurements were conducted per subject, two for each left and right CCA, and the results were averaged. Diameter was assessed at the same location as IMT in real-time M-mode with high-precision vascular echo tracking. Tracking gates were positioned on the CCA near and far walls’ intima-media complexes to automatically monitor wall motion and calculate diameter changes during heart cycles. Four video loops, two for each left and right CCA, were saved from at least five heart cycles. Parameters were computed as the average values of the four measurements. Two experienced investigators performed all assessments; the coefficient of variation for IMT was 4.79%. For further methodological details, see.^23^

IDR was calculated as the ratio between IMT and diameter^37^:

$${{{\rm{IDR}}}}=\frac{{{{\rm{IMT}}}}}{{{{\rm{diameter}}}}}$$

Tensile stress was calculated as follows^30^:

$${{{\rm{Tensile}}}}\; {{{\rm{stress}}}}={{{\rm{MAP}}}} * \frac{{{{\rm{diameter}}}}}{2 * {{{\rm{IMT}}}}}\left[{{{\rm{mmHg}}}}\right]$$

and converted into the unit of [kPa].

#### Cardiorespiratory fitness

CRF was measured using the PACER test from the FITNESSGRAM® test battery. The PACER is a multistage fitness test to assess aerobic capacity. The children run back and forth a distance of 20 meters at a predetermined pace, which is set by an audio signal that increases in speed every minute. The number of achieved laps is counted.^38^ All participants received standardized test instructions and were tested individually by qualified staff. The reliability of repeated test administration for the FITNESSGRAM^®^ PACER test is reported with a κ coefficient of 0.64.^39^ The results of the PACER test were converted into the relative peak oxygen uptake capacity (VO_2_peak), given in ml*kg^−1^*min^−1^, consistent with the currently implemented model in the FITNESSGRAM® software^40,41^:

$${relative}\, {{{{\rm{VO}}}}}_{2}{{{\rm{peak}}}}=45.619+0.353 * {{{\rm{PACER}}}}-1.121 * {{{\rm{Age}}}}\left[\frac{{{{\rm{ml}}}}}{{{{\rm{kg}}}} * {{\mathrm{min}}}}\right]$$

with PACER in laps and age in years.

Relative VO_2_peak was converted to absolute VO_2_peak:

$${absolute}\, {{{{\rm{VO}}}}}_{2}{{{\rm{peak}}}}=\frac{{{{{\rm{relative}}}}\, {VO}}_{2}{{{\rm{peak}}}}}{1000}* {body\; mass}\left[\frac{L}{\min }\right]$$

As multiple authors such as Tanner,^42^ Katch and Katch,^43^ Nevill,^44^ and more recently Armstrong and Welsman^45,46^ criticized the simple ratio-scaling of VO_2_peak to body mass as an inappropriate adjusting method (for more details, see ibid.), we performed an allometric scaling. We used the following approach^44,45^ to calculate the allometrically scaled VO_2_peak:

$${{scaled\; VO}}_{2}{peak}=\frac{{absolute}\, {{VO}}_{2}{peak}* 1000}{{\left({body\; mass}\right)}^{b}}\left[\frac{{ml}}{\min * \, {{kg}}^{b}}\right]$$

where absolute VO_2_peak is given in L/min, body mass is expressed in kg, and b is the allometric exponent. The latter was determined with a linear regression analysis using log-transformed data, with log(absolute VO_2_peak) as the dependent variable and log(body mass) as the independent variable. The slope of the regression line was used as the allometric exponent. In the following sections, “VO_2_peak” refers to the allometrically scaled VO_2_peak.

### Statistical analyses

Data were analyzed with the statistical software Rstudio (Version 2023.03.0 + 386, 2022 by Posit Software, PBC, Boston, Massachusetts) using the packages *dplyr*, *car*, *lm.beta*, and *mediation*. After testing for normal distribution, we calculated mean and standard deviation (SD), or median and interquartile range (IQR). Afterward, we assessed sex differences with an independent two-sample t-test or a Mann-Whitney U-test.

The following analyses were performed separately for girls and boys. We computed multiple linear regression models to test the influence of CRF on vascular parameters with VO_2_peak and BMI as independent variables and IMT, diameter, IDR, or tensile stress as the dependent variables. We adjusted the IMT, diameter, and IDR models for age and MAP and the model for tensile stress for age. Further, we calculated a mediation analysis to investigate CRF’s direct and indirect BMI-mediated effects on vascular parameters. VO_2_peak displayed the independent variable, BMI the mediator, and IMT, diameter, IDR, or tensile stress the dependent variables, respectively. We controlled the IMT, diameter, and IDR models for age and MAP and the tensile stress model for age. We tested the significance of the indirect effect using bootstrapping procedures. We computed unstandardized indirect effects for each of the 1'000 bootstrapped samples.^47^ A *p*-value of <0.05 was defined as statistically significant.

## Results

### Study population characteristics

In total, we examined 1017 schoolchildren (534 girls). We had to exclude 320 participants (158 girls) due to incomplete baseline data assessment (missing IMT (*N* = 264), diameter (*N* = 51), SBP (*N* = 1), or PACER (*N* = 4)). Thus, complete data were available for 697 participants (376 girls). For the characteristics of the study population, see Table 1. In total, 46 children were obese (27 girls), 50 were overweight (20 girls), and 601 (329 girls) were of normal weight. In comparison to boys, girls were significantly older (*p* < 0.001), taller (*p* = 0.005), and had a higher body mass (*p* = 0.021). BMI, SBP, DBP, and MAP did not differ between girls and boys. Boys had a greater diameter (*p* < 0.001), higher tensile stress (*p* = 0.001), and a lower IDR (*p* < 0.001) compared to girls; the IMT was equal. Boys accomplished more PACER laps than girls (*p* = 0.001), but the absolute values of VO_2_peak were identical. However, after allometric scaling of VO_2_peak, girls had a significantly higher VO_2_peak than boys (*p* < 0.001). The calculated exponent for allometric scaling of VO_2_peak was 0.87 for girls and 0.92 for boys.

**Table 1:** Characteristics of the study population.

	Total	Girls	Boys
	(*N* = 697)	(*N* = 376)	(*N* = 321)
age (years)^†^	12.0 (10.6–14.1)	12.4 (10.7–14.4)	11.6 (10.4–12.9)
height (cm)**	153.5 (142.7–163.5)	156.5 (144.0–164.0)	150.5 (140.5–161.5)
body mass (kg)*	43.8 (34.4–54.1)	46.0 (35.6–54.9)	41.5 (33.5–53.1)
BMI (kg/m^2^)	18.3 (16.4–20.8)	18.6 (16.6–20.8)	18.0 (16.3–20.8)
SBP (mmHg)	116 (110–122)	116 (110–122)	115 (110–122)
DBP (mmHg)	68 ± 8	68 ± 8	68 ± 8
MAP (mmHg)	84 (79–89)	84 ± 8	84 ± 8
IMT (mm)	0.46 ± 0.03	0.46 ± 0.03	0.46 ± 0.03
diameter (mm)^†^	5.46 (5.15–5.80)	5.37 (5.08–5.69)	5.60 ± 0.49
IDR^†^	0.085 (0.079–0.091)	0.087 (0.080–0.093)	0.083 (0.077–0.090)
tensile stress (kPa)^†^	66.0 (60.2–72.2)	65.4 ± 8.6	67.7 ± 9.4
PACER (laps)**	31 (22–40)	29 (21–38)	34 (22–43)
absolute VO_2_peak (L/min)	1.9 (1.5– 2.3)	1.9 (1.5–2.3)	1.9 (1.5–2.3)
allometrically scaled VO_2_peak (ml/kg^b^*min)^†^	67.2 ± 7.4	68.7 (65.6–73.4)	63.5 (60.0–68.8)

Results are expressed as mean ± standard deviation or median and interquartile range in parenthesis.

*BMI* body mass index, *DBP* diastolic blood pressure, *IMT* intima-media thickness, *IDR* intima-media thickness:diameter-ratio, *MAP* mean arterial blood pressure, *SBP* systolic blood pressure, *VO2peak* peak oxygen uptake capacity.

^b^allometric exponent; *p ≤ 0.05, **p ≤ 0.01, †*p* ≤ 0.001.

### Association of CRF with IMT, diameter, IDR, and tensile stress

In the multiple regression analyses for girls, VO_2_peak was positively associated with both IMT (*β*_stand_ = 0.171, *p* < 0.001) and diameter (*β*_stand_ = 0.173, *p* = <0.001), see Table 2. VO_2_peak was not significantly associated with IDR or tensile stress. BMI was positively associated with diameter (*β*_stand_ = 0.537, *p* < 0.001) and tensile stress (*β*_stand_ = 0.319, *p* < 0.001), but negatively with IDR (*β*_stand_ = −0.326, *p* < 0.001).

**Table 2:** Multiple regression analysis of IMT, diameter, IDR, and tensile stress with the independent variables VO_2_peak and BMI for girls.

models		*β*	*β std*.
IMT^a^ F (4371) = 8.99^†^ Adjusted *R*^2^ = 0.08	VO_2_peak^†^	0.001	0.171
	BMI	0.001	0.103
Diameter^a^ F (4371) = 35.67^†^; Adjusted *R*^2^ = 0.27	VO_2_peak^†^	0.012	0.173
	BMI^†^	0.062	0.537
IDR^a^ F (3371) = 9.98^†^ Adjusted *R*^2^ = 0.09	VO_2_peak	−0.00003	−0.019
	BMI^†^	−0.001	−0.326
Tensile stress^b^ F (3372) = 13.52^†^ Adjusted *R*^2^ = 0.09	VO_2_peak	0.047	0.036
	BMI^†^	0.708	0.319

*BMI* body mass index, *IMT* intima-media thickness, *IDR* intima-media thickness:diameter-ratio, *MAP* mean arterial blood pressure, *VO*_*2*_*peak* allometrically scaled peak oxygen uptake capacity.

*p ≤ 0.05, **p ≤ 0.01, †*p* ≤ 0.001.

^a^adjusted for age, sex, and MAP.

^b^adjusted for age and sex.

In boys, VO_2_peak was positively associated with IMT (*β*_stand_ = 0.139, *p* < 0.02) and IDR (*β*_stand_ = 0.119, *p* = 0.035) but showed no influence on diameter or tensile stress, see Table 3. BMI was positively associated with IMT (*β*_stand_ = 0.169, *p* = 0.006), diameter (*β*_stand_ = 0.381, *p* < 0.001), and tensile stress (*β*_stand_ = 0.218, *p* < 0.001), but negatively with IDR (*β*_stand_ = −0.201, *p* = 0.001).

**Table 3:** Multiple regression analysis of IMT, diameter, IDR, and tensile stress with the independent variables VO_2_peak and BMI for boys.

models		*β*	*β std*.
IMT^a^ F (4316) = 5.02^†^ Adjusted *R*^2^ = 0.05	VO_2_peak^*^	0.001	0.139
	BMI^**^	0.002	0.169
Diameter^a^ F (4316) = 18.78^†^; Adjusted *R*^2^ = 0.18	VO_2_peak	−0.002	−0.037
	BMI^†^	0.053	0.381
IDR^a^ F (4316) = 5.81^†^ Adjusted *R*^2^ = 0.06	VO_2_peak^*^	0.00014	0.119
	BMI^**^	−0.0005	−0.201
Tensile stress^b^ F (3317) = 10.7^†^ Adjusted *R*^2^ = 0.08	VO_2_peak	−0.111	−0.089
	BMI^†^	0.579	0.218

*BMI* body mass index, *IMT* intima-media thickness, *IDR* intima-media thickness:diameter-ratio, *MAP* mean arterial blood pressure, *VO*_*2*_*peak* allometrically scaled peak oxygen uptake capacity.

*p ≤ 0.05, **p ≤ 0.01, ^†^*p* ≤ 0.001.

^a^adjusted for age, sex, and MAP.

^b^adjusted for age and sex.

### Mediation analyses

**Fig. 1:**
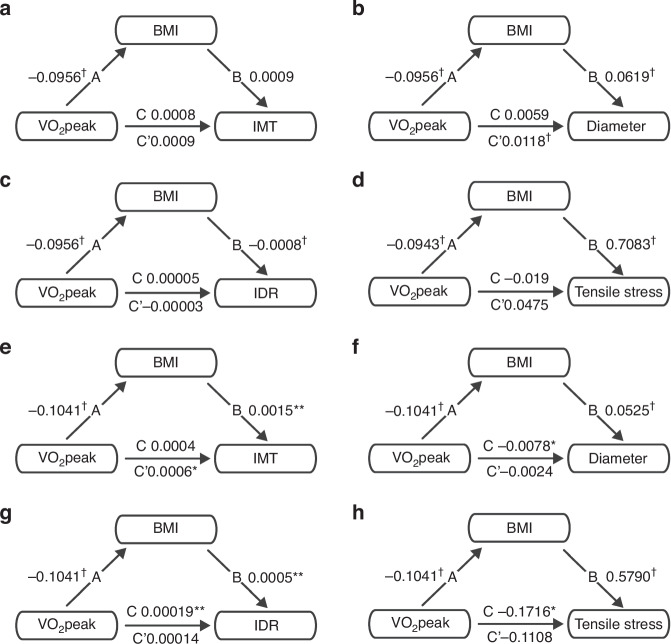
Mediation analysis examining the role of BMI in the relationship between VO_2_peak and vascular parameters. VO_2_peak was used as the independent variable (IV), BMI as the mediator, and IMT for girls (**a**), diameter for girls (**b**), IDR for girls (**c**), tensile stress for girls (**d**), IMT for boys (**e**), diameter for boys (**f**), IDR for boys (**g**), and tensile stress for boys (**h**) as the dependent variable (DV), respectively. Paths A and B reflect the mediation effects; path A describes the impact of the IV on the mediator, and path B describes the effect of the mediator on the DV, controlling for the IV. Path C reflects the total effect of the IV on the respective DV. Path C’ represents the relationship between the IV and DV without considering potential mediators, ergo the direct effect. *p ≤ 0.05, **p ≤ 0.01, ^†^*p* ≤ 0.001.

In the mediation analyses, the average direct effect (ADE) reflects the direct, and the average causal mediation effect (ACME) reflects the indirect effect. The results for girls are shown in Table 4, and those for boys in Table 5. Furthermore, to visualize the findings, they are illustrated in Fig. 1.

**Table 4:** Mediation analysis was performed on girls with VO_2_peak as an independent variable, BMI as a mediator, and IMT, diameter, IDR, and tensile stress as dependent variables, respectively.

	Estimate	CI Lower	CI Upper
**IMT**			
ACME^*^	−0.0001	−0.0002	0
ADE^†^	0.0009	0.0004	0,0014
Total effect^**^	0.0008	0.0003	0,0013
Proportion mediated^*^	−0.1077	−0.3208	−0,0052
**Diameter**			
ACME^**^	−0.0059	−0.0095	−0.0022
ADE^†^	0.0118	0.0056	0.0182
Total effect	0.0059	−0.0011	0.0131
Proportion mediated	−1.0011	−7.648	5.8003
**IDR**			
ACME^†^	0.00007	0.00003	0.00013
ADE	−0.00003	−0.00016	0.00009
Total effect	0.00005	−0.00009	0.00017
Proportion mediated	1.5469	−13.3957	14.8027
**Tensile stress**			
ACME^†^	−0.0668	−0.1251	−0.0227
ADE	0.0475	−0.0779	0.1841
Total effect	−0.0193	−0.1557	0.1369
Proportion mediated	3.4593	−9.0623	14.7758

*ACME* average causal mediation effect, *ADE* average direct effect, *BMI* body mass index, *IMT* intima-media thickness, *IDR* intima-media thickness:diameter-ratio, *VO*_*2*_*peak* allometrically scaled peak oxygen uptake capacity.

*p ≤ 0.05, **p ≤ 0.01, ^†^*p* ≤ 0.001.

**Table 5:** Mediation analysis was performed on boys with VO_2_peak as an independent variable, BMI as a mediator, and IMT, diameter, IDR, and tensile stress as dependent variables, respectively.

	Estimate	CI Lower	CI Upper
**IMT**			
ACME*	−0.0002	−0.0003	0
ADE*	0.0006	0.0001	0.001
Total effect	0.0004	0	0.0009
Proportion mediated	−0.3724	−2.7099	0.754
**Diameter**			
ACME^†^	−0.0055	−0.009	−0.0027
ADE	−0.0024	−0.0085	0.0034
Total effect*	−0.0078	−0.0151	−0.0011
Proportion mediated*	0.6984	0.3072	2.6769
**IDR**			
ACME^†^	0.00005	0.00002	0.0001
ADE	0.00014	0	0.00026
Total effect^**^	0.00019	0.00006	0.00031
Proportion mediated^**^	0.2725	0.0865	1.0064
**Tensile stress**			
ACME^†^	−0.0608	−0.1088	−0.0246
ADE	−0.1108	−0.2425	0.0244
Total effect^**^	−0.1716	−0.3008	−0.0446
Proportion mediated^**^	0.3542	0.1153	1.6558

*ACME* average causal mediation effect, *ADE* average direct effect, *BMI* body mass index, *IMT* intima-media thickness, *IDR* intima-media thickness:diameter-ratio, *VO*_*2*_*peak* allometrically scaled peak oxygen uptake capacity.

*p ≤ 0.05, **p ≤ 0.01, ^†^*p* ≤ 0.001.

### Effect of VO_2_peak on IMT, mediated by BMI

In girls, we found a positive direct effect (ADE: 0.0009, *p* = 0.04) but a negative indirect effect (ACME: −0.0001, *p* < 0.001) of VO_2_peak on IMT. The total effect was positive and statistically significant (0.0008, *p* = 0.002), indicating that the positive direct effect outweighed the negative indirect effect mediated by BMI. The proportion mediated was negative (−0.11, *p* = 0.042), suggesting further that the indirect effect suppresses the total effect rather than mediating it, reflecting a competitive mediation. In boys, similar results were found with a positive direct effect (ADE: 0.0006, *p* = 0.014) and a negative indirect effect (ACME: −0.0002, *p* = 0.02), indicating a competitive mediation. The total effect was positive but was not statistically significant.

### Effect of VO_2_peak on diameter, mediated by BMI

In girls, VO_2_peak displayed a positive direct effect (ADE: 0.0118, *p* < 0.001) but a negative indirect effect (ACME: −0.0059, *p* = 0.002) on diameter. These opposing effects resulted in a non-significant total effect. In boys, only the indirect effect was significant (ACME: −0.0055, *p* < 0.001) and thereby resulted in a statistically significant negative total effect (−0.0078, *p* = 0.024). The proportion mediated was 0.70 and statistically significant (*p* = 0.024), indicating that the indirect effect accounts for a substantial portion of the total effect.

### Effect of VO_2_peak on IDR, mediated by BMI

We found a positive indirect effect of VO_2_peak on IDR in girls and boys (girls: ACME: 0.00007, *p* < 0.001; boys: ACME: 0.00005, *p* < 0.001); the direct effect was not significant in either sex. The total effect was positive in both groups but reached significance only in boys (0.00019, *p* = 0.002), representing an ‘indirect-only mediation’. The proportion mediated estimate was 0.27 and was statistically significant (*p* = 0.006), suggesting an essential contribution to the total effect of VO_2_peak on IDR in boys.

### Effect of VO_2_peak on tensile stress, mediated by BMI

The indirect effect of VO_2_peak on tensile stress was negative and statistically significant for both girls and boys (girls: ACME: −0.0668, *p* < 0.001; boys: ACME: −0.0608, *p* < 0.001). The direct effect did not reach significance, either for girls or boys. The total effect was negative in both groups but was only significant in boys (−0.1716, *p* = 0.006) with a proportion mediated of 0.35 (*p* = 0.006). The latter indicates again that the indirect effect accounts for a substantial portion of the total effect and represents an ‘indirect-only mediation’.

## Discussion

Of our healthy German schoolchildren aged 8-17, 6.6% were obese, 7.2% were overweight, and the majority, comprising 86.2%, fell within the normal weight range. This distribution is similar to that of the KIGGS study, a study on the health of children and adolescents in Germany conducted between 2003 and 2006.^48^ Regarding the vascular parameters, the values for IMT with a mean of 0.46 ± 0.03 mm and for diameter with a median of 5.46 (5.15–5.80) mm align with existing literature,^49^ even though studies with slightly different values also exist.^50–52^ Our participants’ FITNESSGRAM^®^ PACER test scores were comparable to those published in the literature for healthy elementary school children.^53^

The first main finding of our study was that VO_2_peak, a measure of CRF, was positively associated with IMT and diameter. This result suggests that the arterial wall thickens with increasing CRF, and the vessel diameter extends. The latter finding was only significant in girls. Secondly, our study provided more profound insights into the above-described relationship by individually examining CRF’s direct and indirect BMI-mediated effects on vascular parameters. We found that the direct and indirect effects often counteracted each other, indicating a complex interplay between CRF, BMI, and the vasculature. Higher BMI was linked to increased IMT and larger vessel diameter. The inverse relationship between CRF and BMI mitigated these BMI-related vascular alterations. The overall impact of CRF on vascular parameters varied depending on whether the direct positive or indirect attenuating effect was superior: positive with IMT in girls, non-significant with IMT in boys and diameter in girls, or negative with diameter in boys. These findings highlight the importance of considering direct and indirect pathways separately when interpreting CRF’s effect on vascular structure.

Interestingly, VO_2_peak showed no direct association with IDR or tensile stress. This finding suggests that the vessel remodels homogeneously as CRF improves and hemodynamic forces overall remain stable. In contrast, higher BMI was associated with decreased IDR and increased tensile stress, indicating unbalanced remodeling and disrupted hemodynamics. Once again, CRF mitigated these BMI-dependent effects due to its inverse relationship with BMI.

In line with our findings, previous studies revealed a higher IMT, an increased brachial artery diameter,^21,54^ and decreased tensile stress^20^ in athletes such as professional football players^20,21^ or wrestling players^22^ compared to controls. Nevertheless, VO_2_peak was partly lower in athletes than controls.^21^ Contradictory results also exist, demonstrating a reduced IMT^19,54–56^ and diameter^56^ or unchanged IMT,^57^ diameter,^20,22^ and IDR^22^ in athletes and fit subjects compared to controls. However, some of these investigations lacked sufficient BMI control. Given the results of our mediation analyses with contradictory direct and indirect BMI-related effects, neglecting body composition in the analysis harbors the risk of distorting CRF’s impact on vessel structure and might, at least partially, explain the differences compared to our study.

Several aspects should be considered when interpreting the relationship between CRF and vascular parameters. The IMT consists of two layers: the intima and the media. Alterations in both layers can contribute to an increase in IMT.^36^ In cases of manifest atherosclerosis, the increase in IMT is due to an inflammatory proliferation of the intima layer.^58^ Regarding exercise, changes in the vessel wall may instead occur due to hemodynamically triggered alterations in the media layer.^59^ Exercise is known to alter hemodynamic forces, as it increases blood flow and, with this, shear stress.^60^ In the short term, vessels may respond with functional adaptations, like increased bioavailability of vasodilator molecules. With continued training, these functional changes can be surpassed by anatomical adjustments: enlarging its diameter enables a vessel to restore shear stress structurally.^61,62^ But, a larger diameter, in turn, increases tensile stress. The arterial wall might compensate for this increase in tensile stress with a thickening driven by vascular smooth muscle cells (VSMCs) of the media layer.^59^ Thus, the CRF-associated thickening of the arterial wall and the diameter enlargement in our study population might reflect physiological adaptation to altered hemodynamic forces due to regular bouts of exercise rather than subclinical atherosclerosis. Affirming this suggestion, CRF did not directly influence IDR and tensile stress despite its impact on vessel wall and diameter, indicating homogenous remodeling and balanced hemodynamics with increasing CRF.

In contrast, this theory of physiological adaptation to hemodynamic factors may not adequately explain the vascular changes associated with BMI. BMI also triggers hemodynamic changes through increased blood flow and elevated shear stress.^63^ Following the hemodynamic cascade, one would expect the diameter to enlarge, the tensile stress to elevate, and ultimately, the arterial wall to thicken to restore altered hemodynamic forces to baseline values.^64^ Indeed, our findings revealed that a higher BMI is associated with increased IMT and diameter. But, unlike with higher CRF, IDR decreased, and tensile stress increased with higher BMI. This result suggests that the vessel diameter’s enlargement exceeds the vascular wall’s thickening, potentially preventing the restoration of hemodynamic forces. Thus, CRF- and BMI-associated vascular alterations appear to affect the vascular structure differently, although both lead to increased IMT and diameter.

Obesity, in our study reflected by higher BMI, is considered a low-grade inflammatory disease.^65^ Previous research has primarily postulated an associated increase in IMT—explained by inflammation-induced intima proliferation.^8,32,66–68^ However, in a study on over 4000 healthy children, it was the fat-free mass, reflecting physiologic growth, not the fat mass, reflecting obesity, that was associated with an increase in IMT.^69^ The authors used an ultra-high frequency ultrasound device to distinguish the individual contributions of the intimal and the medial layers separately. Whereas a higher fat mass did neither evoke intimal nor media layer adaptation, an increased fat-free mass induced medial layer proliferation.^69^ This result suggests that physiological processes like growth lead to an increase in IMT through media adaptation. The children of Chiesa’s study showed equal increases in IMT and diameter with unchanged IDR and balanced tensile stress - similar to the children with higher CRF in our study. Interestingly, the children with higher fat mass showed reduced IDR and increased tensile stress,^69^ which mirrors our results for increasing BMI.

These alterations -excessive diameter enlargement accompanied by reduced IDR and increased tensile stress- resemble alterations well-known from another vascular pathology: aneurysm formation. In aneurysms, the VSMCs of the vascular wall are dysfunctional.^70^ These defective VSMCs culminate in a “media dysfunction” and cause a destabilized wall, provoking a dilated and unstable vessel.^70^ In adults, Rodriguez-Macias et al. found that subjects with a diagnosis of CVD, coronary heart disease, myocardial infarction, or stroke had not only a significantly thicker intima layer but, surprisingly, a thinner media layer than healthy controls.^58^ This observation suggests that CVD risk factors are not only associated with an inflammatory-induced increase in the intima layer but, in addition, with a (possibly also inflammatory-triggered) decrease in media thickness. Thus, a “media dysfunction” potentially also explains the BMI-associated insufficient wall thickening in the present study, which led to a decreased IDR and increased tensile stress. In line, a previous investigation comparing obese and normal-weight children revealed an increased diameter, elevated tensile stress, and a higher arterial stiffness in obese subjects without differences in IMT between both groups.^32^ On the contrary, Weberruß et al. found that children with higher CRF showed lower arterial stiffness despite an increased IMT^24^—perhaps due to beneficial adaptations of VSMCs in the media layer.

Taken together, a higher CRF may lead to beneficial media layer adaptation, while obesity, as an inflammatory disease and cardiovascular risk factor, may be accompanied by medial dysfunction. However, these suggestions remain hypothetical and warrant further investigation, primarily through longitudinal studies using ultra-high frequency ultrasound to distinguish intimal and medial adaptations.

The present study has some limitations. First, we did not assess and control for further potentially confounding factors of IMT and diameter, such as familial hypercholesterolemia,^71^ increased concentrations of serum c-reactive protein,^72^ serum uric acid,^73^ plasma total homocysteine,^74^ and cholesterol level,^75^ as well as pubertal maturation,^76^ maternal obesity,^77^ or birth-related issues.^78,79^ Second, the trainability of VO_2_peak, the main parameter for determining CRF, is genetically determined up to 50%^80^ and may interfere with the interpretation of arterial adaptation caused by regular exercise. Furthermore, the lack of information on the maturity status of our participants is a severe limitation, as maturity is associated with VO_2_peak in children.^81^ Comparability to previous study results may also be limited, as we assessed CRF with the test battery FITNESSGRAM^®^. In contrast, others used treadmill^21^ or cycle tests^56,57^ for assessing CRF. Armstrong et al. argued that the prediction of VO_2_peak from a shuttle run test might not be sufficiently accurate and that comparisons between different assessments of VO_2_peak are too imprecise.^46^ Nevertheless, the FITNESSGRAM^®^ test has good validity and is an easily accessible test method in a school-based setting.^82^ Finally, due to its cross-sectional design, the findings of our study are descriptive, and further longitudinal studies would be desirable to assess CRF-induced vascular adaptations directly.

In conclusion, CRF-associated remodeling might be homogenous and reflect physiological adaptation rather than subclinical atherosclerosis. On the contrary, BMI-associated changes may depict unbalanced remodeling accompanied by disturbed hemodynamic forces - potentially adverse alterations that may be alleviated by higher CRF, highlighting its significant role in the cardiovascular health of children.

## Data Availability

The data presented in this study are available upon reasonable request from the corresponding author.
